# Prophylactic temporary abdominal aorta balloon occlusion in women with placenta previa accretism during late gestation

**DOI:** 10.1097/MD.0000000000008681

**Published:** 2017-11-17

**Authors:** Zhongyuan Qiu, Jifen Hu, Jianbo Wu, Lihong Chen

**Affiliations:** a Obstectic & Gynecology, Fujian Medical University Union Hospital; b Obstectic & Gynecology, the Third Affiliated Hospital of Fujian Medical University; c Obstetrics & Gynecology, the First Affiliated Hospital of Fujian Medical University, China.

**Keywords:** balloon occlusion, cesarean section, hemorrhage, hysterectomy, placenta previa accretism

## Abstract

**Background:**

To evaluate the clinical efficacy of prophylactic temporary balloon occlusion of the abdominal aorta in patients with placenta previa accretism during cesarean section.

**Methods:**

Twenty-three consecutive patients, prenatally confirmed with placenta previa accretism were retrospectively analyzed in our center from August 2012 to October 2014. All 23 subjects underwent cesarean section with prophylactic balloon occlusion of the abdominal aorta.

**Results:**

All of the 23 subjects experienced singleton pregnancies leading to the birth of live infants. Of these subjects, the following problems were diagnosed: placenta accrete (n = 10), placenta increte (n = 10), and placenta precrete (n = 3). Mean intraoperative hemorrhage was 1170.0 mL. Fifteen patients received red blood cell transfusion with a mean transfusion volume of 2.3 units. The incidence of hysterectomy was 21.74% (5/23) with blood loss ranging from 2000 to 5000 mL (mean 3360.0 mL). One complication encountered in this retrospective study was lower extremity arterial thrombosis. Eighteen patients were followed-up by telephone to 14 months following discharge, all babies were noted to be healthy.

**Conclusion:**

Prophylactic abdominal aorta balloon occlusion (ABO) was relatively safe in the treatment of patients with placenta previa accretism. This approach could represent a key aspect in a multidisciplinary algorithm in reducing hemorrhage in abnormal placentation.

## 1 Introduction

Abnormal attachment of the placenta includes conditions known as placenta accreta, increta, and percreta, in which chorionic villi invade into the myometrium. The term placental accretism is very popular and is used to describe most situations. Severe obstetric hemorrhage, massive transfusions, and significant maternal morbidity, are commonplace in placenta accrete, which represents the predominant cause of peripartum hysterectomy.^[1]^ Placenta accrete is reported to occur in <1:700,000 to 1:500 pregnancies.^[2]^ However, owing to the increasing rate of cesarean delivery over the last few decades, the number of reported cases with abnormal attachment of the placenta has increased 10-fold.^[3]^

The accepted treatment for placenta accretism is cesarean hysterectomy, but this comes with a significant risk of catastrophic bleeding during surgery. Bilateral hypogastric, or uterine artery ligation, are the normal surgical techniques deployed to stop such bleeding. Nevertheless, extensive collateral circulation is likely to reduce the effect of hemostasis, particularly during pregnancy. Blood flow can be abundant in cases where the placenta covers the cervix and invades adjacent tissues, with consequential high rates of morbidity and mortality.

Since the introduction of prophylactic temporary abdominal aorta balloon occlusion (ABO) as a treatment for postpartum hemorrhage in 1979, trans-catheter artery embolization has increasingly become a key technique for reducing blood loss and reducing the rates of hysterectomy compared with conventional techniques.^[4]^ However, existing literature, which predominantly includes case reports, and studies with small sample sizes, do not appear to be robust enough to provide a thorough evaluation of this technique, particularly as earlier studies were lacking in statistical power.^[5–7]^ As a consequence, in order to refine the treatment of peripartum hemorrhage, it is important that appropriately designed studies determine an optimized protocol for patient selection, and to determine the appropriate position of the predelivery prophylactic pelvic artery balloon occlusion.

Antenatal diagnosis of placenta accretism is crucial for planning the appropriate treatment management plan to reduce morbidity and mortality. The use of ultrasound Doppler analysis and magnetic resonance imaging (MRI) in high-risk populations have permitted scheduled cesarean hysterectomy and multidisciplinary presurgical planning, and as a result, both maternal and neonatal outcomes have been significantly improved.^[8]^ However, the prophylactic use of temporary balloon occlusion of the abdominal aorta in the treatment of placenta accreta, has yet to be evaluated in a robust manner, and may provide clinicians with improved therapeutic options.^[9,10]^ The abdominal aorta balloon has been used successfully for the treatment of pelvic fractures and tumors for some time now.^[11,12]^ In the present study, we present our experience of prophylactic temporary balloon occlusion of the abdominal aorta in a cohort of 23 patients with suspected placenta accreta, and describe the outcome of hemorrhage control as an alternative to minimize bleeding.

## 2 Materials and methods

**Figure 1:**
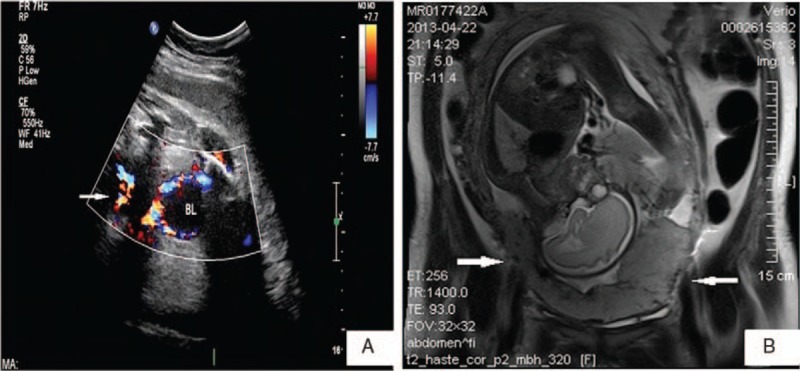
A. Ultrasonagraphy showing multiple hypoechoic spaces on the placenta (denoted by arrows). B. Axial T1-weighted MRI image showing complete placenta previa with invasion into the anterior/posterior myometrium of the uterus, suggestive of placenta increta (denoted by arrow).

Clinical records of 23 women who had abdominal aorta balloons inserted prior to cesarean section were reviewed and analyzed retrospectively in the Department of Gynecology and Obstetrics at The First Affiliated Hospital of Fujian Medical University between August 2012 and October 2014. Our local Institutional Review Board approved the study, and all recruited patients provided informed consent for the subsequent use of their clinical data for research purposes. Diagnosis of placenta previa accretism was based on previa totalis and prior cesarean delivery with accretism, ultrasound findings, and MRI scans (Fig. 1). A multidisciplinary team was scheduled for disease management, including the departments of obstetrics, anesthesiology, interventional radiology, neonatology, and the blood bank.

**Figure 2:**
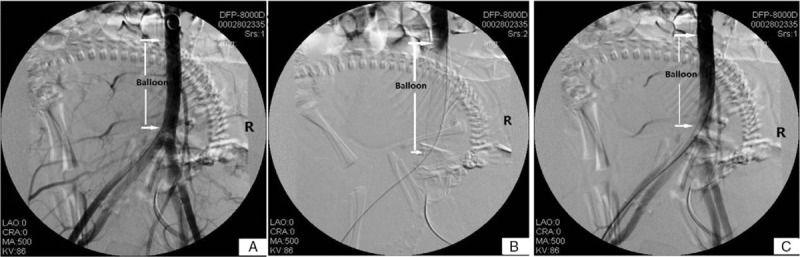
A. Positioning of the occlusion balloon catheter above the bifurcation of the abdominal aorta. B. Inflation of the balloon to ensure adequate vessel occlusion. C. Deflation of the balloon to permit resumption of blood flow.

Prior to surgery, patients and their families were appropriately informed of surgical and procedural risks. The radiologist chose to use a temporary occlusive balloon in the infra-renal abdominal aorta based on whether there was sufficient collateral circulation or not. On the day of scheduled cesarean delivery, the patient was sent to the angiography suite before the operation and the right femoral artery was punctured using the standard percutaneous Seldinger technique. Local regional anesthesia was provided by lidocaine via the placement of a 5-French guide catheter sheath (Bard Co. Ltd., NJ). Under fluoroscopy guidance, the occlusion balloon catheter (Bard Co. Ltd., American) was then positioned above the bifurcation of the abdominal aorta without contrast medium. Once placed, the balloons were temporarily inflated to ensure adequate vessel occlusion, then deflated, fixed with skin sutures, and covered by surgical tape (Fig. 2). Fluoroscopy time ranged from 2 to 2.5 minutes under a regimen of 10 mGy dosimetry fetal exposure.

In the operating theatre, cesarean delivery was selectively carried out in the upper uterine segment, thus avoiding the placenta and its edge. Once the umbilical cord had been cut, the occlusion balloons were inflated to the size needed to occlude the aorta, and then deflated after a period of 25 to 30 minutes. This procedure was repeated when hemostasis of the placenta bed had been successfully achieved. Meanwhile, additional uterotonics (oxytocin, misoprostol, and carboprost) were administered, along with surgical ligation of uterine arteries or B-Lynch suture, as and when required. If significant hemorrhaging occurred and could be controlled, then the balloon was reinflated and hysterectomy performed immediately. Considering the risk of delayed pastpartum hemorrhage and infection, we did not leave the placenta in situ. Patients were nursed flat to prevent distortion of the femoral sheath and subsequent femoral artery thrombosis. If the postoperative course was uneventful, endovascular instruments were removed within 24 hours, and manual compression was firmly applied over the puncture sites for at least 20 minutes.

All procedures were performed by an experienced radiologist and a veteran gynecologist, while general anesthesia was monitored by a senior anesthesiologist. Primary outcome measures included estimated bleeding volume, the volume of blood transfused, and the time taken for surgery to be completed. We also recorded the frequency, nature, and severity of complications related to the balloon catheters.

## 3 Results

All of the patients recruited to this study (n = 23) were diagnosed antenatally with placenta accretism by ultrasound and MRI, and the diagnosis confirmed clinically by surgery or histological examination. Demographic characteristics and perioperative details of our entire patient cohort are given in Table 1. All cesarean sections were carried out under spinal-epidural anesthesia, and 13 patients were given general anesthesia after the infant was born.

**Table 1:**
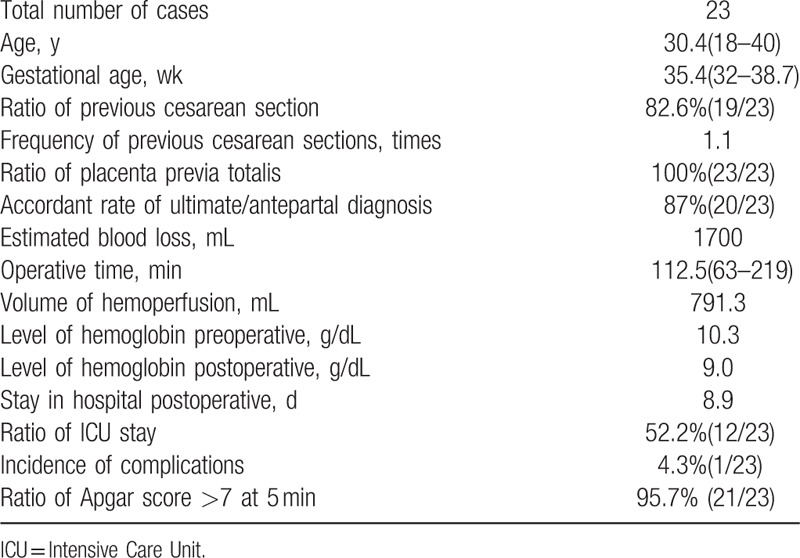
Demographic characteristics and perioperative details.

Eighteen patients experienced a favorable outcome, in which the uterus was preserved and blood loss was only 500 to 2500 mL, with a mean of 1238.9 mL. Poor outcomes arose in 5 patients with placenta precreta, resulting in hysterectomy. The overall rate of hysterectomy during our study was 21.74%. Of the 5 patients with poor outcomes and experiencing hysterectomy, 3 exhibited significant adhesions to the bladder, or the parietal peritoneum. Hysterectomy was also associated with increased intraoperative blood loss, with a mean intraoperative blood loss of 3360 mL (range 2000–5000 mL). Table 2 shows data regarding estimated blood loss and blood transfusion.

**Table 2:**
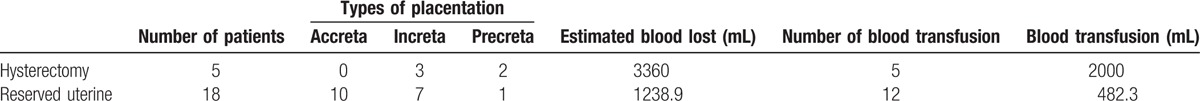
Blood loss and blood transfusion for both fertility preserved and removed.

One case of lower extremity arterial thrombosis was recorded, which received anticoagulant therapy. This occurred 48 hours after surgery and may have been directly associated with the interventional procedure. There were no other instances of relevant complications, such as the formation of hematoma or pseudoaneurysm. Twelve of our 23 patients were sent to the intensive care unit for observation but did not require any treatment.

After discharge, we followed-up 18 patients by telephone for a 14 months period. One single case required a second surgical intervention 15 days post-delivery due to a pelvic abscess. During the follow-up period, the patient diagnosed with lower extremity arterial thrombosis had completely returned to normal. All babies were healthy and developing normally.

## 4 Discussion

Postpartum hemorrhage is the predominant cause of maternal morbidity and mortality in placenta previa accretism. The definitive treatment for most cases of this condition is to carry out cesarean hysterectomy. While prophylactic temporary balloon occlusion participates in the multidisciplinary algorithm for postpartum hemorrhage, there is still some concern with the existing literature describing this mode of treatment, particularly with regard to statistical power. However, our study highlighted that abdominal ABO could reduce bleeding during cesarean section in patients with placenta previa accretism.

To our knowledge, a variety of methods have been deployed to reduce blood loss during cesarean section, including B-lynch suture, perioperative prophylactic trans-arterial artery balloon occlusion, and embolization, uterine tamponade balloon, and ligation of the uterus or internal iliac artery. A number of previous papers^[9,13–15]^ describe balloon occlusion most commonly being placed in a distal position, such as the branch of the internal and common iliac arteries. Occlusion of the internal iliac artery with a balloon is a temporary and less invasive interventional approach, and is very successful in some cases. However, published results are unfortunately not consistent. In a case-control comparison, Shrivastava et al^[15]^ showed that inflation of a balloon in the internal iliac artery balloon at the time of delivery failed to reduce intraoperative hemorrhage in any of the 19 patients he reported. Further data reported by Levine et al^[14]^ and Bodner et al^[5]^ also demonstrated that prophylactic balloon occlusion in the internal iliac artery prior to hysterectomy did not provide any benefit in reducing blood loss. Within a group of 17 women with placenta percreta, Clausen and Langhoffroos^[16]^ reported 8 patients undergoing cesarean hysterectomy; the remainder of the cohort experienced local placenta resection with blood loss estimated to be 4050 mL. These authors considered that balloon occlusion of the infra-renal aortic and the common iliac arteries, rather than the internal iliac arteries, will block vascular supply to the placenta accrete from the external iliac arteries.

In our hospital, prophylactic abdominal aorta occlusion was used to manage placenta previa accretism during cesarean section and our experience of this technique may have some benefits. Occlusion of the abdominal aorta can control bleeding more effectively than using a balloon catheter in the iliac artery because of the rich collateral circulation in the pelvis bridging the internal and external iliac arteries.^[17]^ Bell-Thomas et al^[18]^ also described the emergency use of an aortic occlusion catheter to control a massive hemorrhage during Cesarean hysterectomy in a patient with placenta percreta when attempting to identify the flat surface of the placenta. This procedure successfully resulted in a relatively bloodless field. In addition, blood flow from the ovarian and higher lumbar arteries remain unaffected. Moreover, vasospasm can occur relatively simply in small-caliber arteries, and thus lead to placenta hypo-perfusion, antepartum fetal distress, and ultimately require an urgency cesarean section. In the present study, mean estimated blood loss was 1700 mL during the surgical procedure, although in the 5 patients undergoing cesarean hysterectomy, blood loss rose to 3360 mL. In spite of lacking a specific control group, we systematically compared the details of balloon occlusion placed in the abdominal aorta with other groups^[9,10,18–20]^ (Table 3).

**Table 3:**
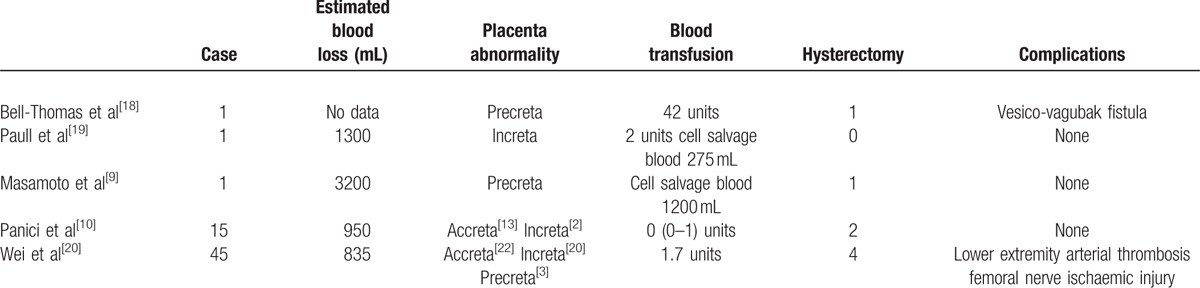
Primary outcomes recorded in the treatment of abdominal balloon occlusion at cesarean section.

Accurate prenatal diagnosis is indispensable for providing affected patients with adequate time for counseling and planning prior to delivery, which are highly likely to contribute to better outcomes. An earlier retrospective review of 66 cases of placenta accrete concluded that undiagnosed incidences of abnormally invasive placenta led to significantly more emergency hysterectomies and mass transfusions, and that prenatal diagnosis of this condition can reduce morbidity.^[21]^ However, diagnosing placenta accrete during the prenatal period remains very difficult. Yi et al^[22]^ proposed the use of interventional radiology in order to reduce the amount of bleeding and maternal morbidity associated with cesarean section. Our study also established that ultrasound Doppler combined with MRI-based diagnosis is very useful in detecting placenta previa accretism, with a reliability rate of almost 90%. Moreover, consultation between multidisciplinary teams is recommended in scheduling cases of cesarean section involving placenta previa accretism, and in our case, involved representatives from perinatology, gynecology, neonatology, radiology, and anesthesiology.

Significant efforts have been attempted to minimize fetal exposure by taking appropriate precautions and by reducing exposure to low-dose radiation during the procedure. However, the precise relationship between fetal fluoroscopy and the potential risk of cancer in children remains uncertain. Brent^[23]^ reported that an exposure of 200 mGy would not increase the rate of congenital malformation, and consequently, the 10 mGy of fluoroscopy used in our present study can be considered to be relatively safe. In reference to documents published by the National Radiological Protection Board,^[24]^ the fetal exposure in our study reached only the level of diagnosis. Technically, it is far easier and quicker to lodge the balloon in the aorta than in smaller arteries because of the shorter theoretical distance, coincident with reduced risk of fetal exposure to radiation.

Complications associated with artery balloon occlusion have been reported in several earlier publications.^[25,26]^ For example, Shrivastava et al^[15]^ reported the rate of complication (15.8%) directly depended upon catheter placement in a 9 years study. In our study, we observed only 1 case of deep venus thrombosis of the lower limb that was directly related to balloon occlusion because she refused to exercise. Sewell et al^[26]^ also reported a popliteal arterial embolus, which occurred during cesarean hysterectomy despite heparin administration and repeated inflation-deflation (every 15 minutes) of a balloon during occlusion of the common iliac artery. As a consequence, prophylactic arterial balloon occlusion may elevate the risk of thrombus in pregnant women. Considering the varying conditions of health care when compared across different institutions, it is difficult to readily evaluate the relative risk of complication in a consistent manner. Nevertheless, the significance of utilizing experienced interventional radiologists must be highlighted, as proficient catheterization and appropriate positioning of the catheter can help prevent complications, at least to a certain degree.

We should, however, highlight several weaknesses in our retrospective study. Firstly, we are unable to draw definitive conclusions as only a limited number of patients were recruited, thus necessitating further study with a larger sample size. Another issue is that our study was retrospective in nature, as with all other studies reported previously. Consequently, there is a very real need for prospective randomized controlled trials. In order to increase scientific rigor, it will be necessary to deploy statistical power calculations in order to select a suitable number of patients for artery balloon occlusion, along with an appropriate control group. This will allow appropriate statistical analysis, and thus provide robust outcomes. In addition, due to the limited number of patients recruited to our present study, it was not possible to compare clinical outcome according to the degree of invasion. Attempts should also be made to leave the placenta in situ, and blood flow in the lower extremities should be monitored during the perioperative period.

In summary, the present study showed that prophylactic abdominal aorta balloon catheters are relatively safe, and represent a feasible treatment for reducing hemorrhage in women diagnosed with placenta previa totalis combined with accretism. However, as yet, there is no convincing evidence that this technique can reduce hysterectomy. Prospective randomized trials are urgently required in order to evaluate further both efficacy and outcome. Given the low incidence of this condition, a polycentric method may permit derivation of an accurate conclusion.

## Acknowledgments

The authors wish to thank their colleagues who participated in the multidisciplinary treatment algorithm at the first Affiliated Hospital of Fujian Medical University.
